# Early Detection of Cerebral Vasospasm Following Aneurysmal Subarachnoid Hemorrhage Using Serum and Cerebrospinal Fluid Biomarkers

**DOI:** 10.7759/cureus.93865

**Published:** 2025-10-05

**Authors:** Naeem ul Haq, Rizwan Ali, Musawer Khan, Muhammad Ishaq

**Affiliations:** 1 Neurosurgery, Bacha Khan Medical College, Mardan, PAK; 2 Neurosurgery, Mardan Medical Complex, Mardan, PAK; 3 Neurosurgery, Mardan medical Complex, Mardan, PAK

**Keywords:** aneurysm, biomarkers, early detection, subarachnoid hemorrhage, vasospasm

## Abstract

Background/introduction

The aim of this study was to understand the effectiveness of particular serum and cerebrospinal fluid (CSF) biomarkers for the early diagnosis of cerebral vasospasm (CV) in patients with aneurysmal subarachnoid hemorrhage (aSAH) and to document any correlation with clinical and radiological outcomes. aSAH carries a high risk of CV and delayed cerebral ischemia, which are leading causes of disability and death. Current diagnostic tools like transcranial Doppler and angiography often detect vasospasm only after clinical manifestation, highlighting the need for early predictive biomarkers. This study investigates the potential of serum and CSF biomarkers, specifically IL-6, ET-1, and S100B, to enable early diagnosis of CV and improve correlation with clinical and radiological outcomes.

Materials and methods

The data were obtained from the Department of Neurosurgery in Mardan Medical Complex Hospital in Mardan, Pakistan, from July 2019 to July 2024. The study included 60 patients aged 18 to 80 years with a diagnosis of aSAH who were admitted within 48 hours of the onset of the disease. Blood and CSF were obtained on days 1, 3, and 7 after ictus. The analyzed biomarkers were interleukin-6 (IL-6), S100 calcium-binding protein B (S100B protein), and endothelin-1 (ET-1). Transcranial Doppler ultrasonography was used to evaluate vasospasm, and computed tomography angiography was used when necessary. SPSS software was used to analyze data, with statistical significance set at p < 0.05. Logistic regression analysis was performed to understand the relationship between biomarker levels and the occurrence of vasospasm.

Results

Radiology confirmed the diagnosis of vasospasm in 35 (58.3%) of the 60 patients with a mean age of 55.4 ± 12.1 years. On day 3, the levels of IL-6 and endothelin-1 were significantly higher in the vasospasm group than in the non-vasospasm group (p = 0.003 and p = 0.012, respectively), while S100B findings were inconsistent. The risk of vasospasm was 2.5 times higher in patients with increased IL-6 values. Early elevation of biomarkers was associated with poor Glasgow Outcome Scale scores at discharge (p = 0.016). Elevated serum IL-6 (>100 pg/mL) and CSF ET-1 (>10 pg/mL) on day 3 were significant predictors of poor outcome, with odds ratios of 3.2 (95% CI 1.4-7.4; p = 0.005) and 2.8 (95% CI 1.2-6.6; p = 0.015), respectively.

Conclusion

Serum and CSF biomarkers, particularly IL-6 and endothelin-1, may help identify CV early after aSAH, even without radiological signs. Incorporating these biomarkers into care could enable earlier treatment and improve outcomes, though multicenter studies are needed to establish standardized protocols.

## Introduction

Intracranial aneurysms are abnormal dilations of cerebral arteries that occur in 3%-5% of the population and involve loss of the internal elastic lamina and alteration of the tunica media [1]. The consequence of a cerebral aneurysm is rupture, with a morbidity and death rate of more than 50%. Of patients with aneurysmal subarachnoid hemorrhage (aSAH), 15% to 35% experience some form of lifelong neurological deficit, cognitive deficit, or personality change [2].

The factors associated with outcomes in aSAH and delayed cerebral ischemia include aneurysmal rebleeding, epileptic seizures, and cerebral vasospasm (CV) [3]. For those who survive a ruptured aneurysm, cerebral ischemia remains the leading cause of disability, affecting 30% of such patients. The condition occurs in approximately 30% of individuals who initially experience the hemorrhage, which typically arises 4 to 10 days after the onset of aSAH. CV has long been recognized as the underlying pathological mechanism of cerebral ischemia. Current evidence suggests that the pathophysiology of cerebral ischemia is considerably more complicated and is multifactorial. Cerebral ischemia occurs during the acute phase of neuronal cell injury [4] and results in loss of cerebral autoregulation, microcirculatory abnormality, thrombosis in small vessels, cortical spreading depolarization, and inflammation in neuronal cells [5].

Diagnostics such as transcranial Doppler (TCD) ultrasound and cerebral angiography allow for the detection of disease. However, these methods usually detect vasospasm after the development of symptoms or the observation of an alteration on a radiograph [6]. TCD and angiography remain the primary diagnostic tools, but they have limitations such as late detection, invasiveness, and the inability to predict CV early.

**Figure 1:**
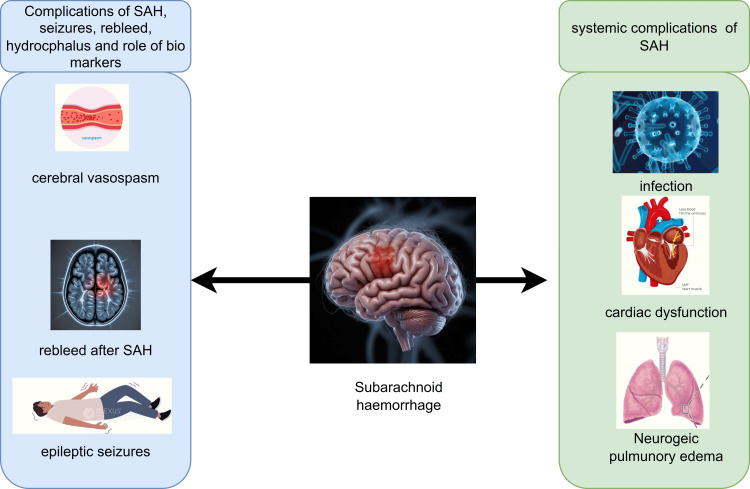
Factors causing poor outcome after aneurysmal subarachnoid hemorrhage. Artwork created by the authors. SAH: subarachnoid hemorrhage.

Serum and CSF biomarkers such as interleukin-6 (IL-6) and endothelin-1 (ET-1) indicate inflammatory and endothelial dysfunction, and they rise days before radiographic or clinical manifestations. This lag reduces the prospect of timely therapeutic intervention and increases the likelihood of negative neurological outcomes. Serum biomarkers are valuable indicators of normal biological processes, pathological processes, and the body’s response to exposure or intervention in aSAH. Serum and cerebrospinal fluid (CSF) biomarkers, especially those based on inflammatory and endothelial dysfunction, have demonstrated potential for detecting giant cell arteritis [7], as shown in Figure 1. Previous methods include the use of IL-6, ET-1, and S100 calcium-binding protein B (S100B protein), which indicate inflammatory cascade activation and neurovascular injury, as biomarkers [8]. Interestingly, the levels of these biomarkers are associated with the development of vasospasm as well as poor clinical outcomes [9].

Several studies have explored the role of S100B and neuron-specific enolase (NSE) in predicting CV and poor outcomes after aSAH. Herrmann et al. [10] demonstrated that elevated S100B levels in the first four days after ischemic stroke correlated with large infarct volumes and poor prognoses. Oertel et al. [11] found that S100B levels increased in SAH patients with CV (as assessed by TCD), while NSE levels decreased, though only the change in S100B levels was associated with poor outcomes. Weiss et al. [12] reported no direct correlation between S100B and CV but confirmed the strong prognostic value of this biomarker for neurological outcomes. Sanchez-Peña et al. [13] observed that increases in S100B specifically predicted ischemic vasospasm and poor outcomes. Moritz et al. [14] found that neither S100B nor NSE correlated with CV, but both were linked to adverse outcomes, with S100B being the more consistent prognostic marker.

These findings suggest that while S100B is a robust predictor of poor outcomes, its relationship to CV remains inconsistent. Thus, there is a need to assess multimodal biomarkers in the management of aSAH [11,14]. Accordingly, the aim of this study was to investigate the role of serum biomarkers and their relationship to CV and evaluate the diagnostic value of sequential plasma and CSF values of IL-6, ET-1, and S100B in predicting the development of early vasospasm. We also compared the trends in the biomarkers with subsequent radiographic and clinical findings in aSAH patients.

## Materials and methods

This study was designed to investigate the association between serum biomarkers and the occurrence of CV and DCI following aSAH. Clinical and demographic data, including age, sex, comorbidities, and severity grading, were documented at admission. Serum biomarker sampling and assessment protocols were carried out in accordance with previously published methodologies, as shown in Table 1, where S100B and NSE have been most consistently studied in relation to CV and DCI. Standardized diagnostic modalities, including TCD, computed tomography (CT), and angiographic evaluation, were used to confirm CV and DCI. Statistical analyses were applied to compare groups, assess correlations, and evaluate the prognostic value of biomarker levels for adverse outcomes.

**Table 1:** Summary of the literature focusing on S100B and NSE as biomarkers after aSAH predictive of CV and/or DCI. NSE: neuron-specific enolase; CV: cerebral vasospasm; DCI: delayed cerebral ischemia; S100B: S100 calcium-binding protein; NE: neurological evaluation; TCD: transcranial Doppler; aSAH: aneurysmal subarachnoid hemorrhage; CT: computed tomography; ICU: intensive care unit.

Study	Serum Biomarker	Sample Collection	CV Assessment Method	CV Correlation	DCI Assessment Method	Negative Outcome
Herrmann et al. [10]	S100B	First 4 days after ischemic stroke	NE	NE	CT (lesion volume)	++ (prognostic)
Oertel et al. [11]	S100B NSE	First 3 days after aSAH	TCD	↑ (increase) ↓ (decrease)	NE	+ (↑ with S100B)
Weiss et al. [12]	S100B	First 8 days after aSAH	TCD + arteriography	− (no correlation)	NE	++ (prognostic)
Sanchez-Peña et al. [13]	S100B	First 15 days after aSAH	TCD + arteriography	↑ in ischemic vasospasm	NE	++ (↑ prognostic)
Moritz et al. [14]	S100B, NSE	Daily during ICU stay	TCD	− (no correlation)	CT	++ (prognostic) + (NSE peak only)

This prospective observational study was conducted in the Department of Neurosurgery at Mardan Medical Complex in Mardan, Pakistan, from July 2019 to July 2024. The factors responsible for poor outcomes are illustrated in Figure 1. We recruited adult patients (≥18 years of age) presenting with confirmed aSAH based on CT angiography within 48 hours of ictus. Blood and CSF measurements were taken on days 1, 3, and 7 following ictus. An enzyme-linked immunosorbent assay (ELISA) kit (Merck, Billerica, Massachusetts) was used to quantify serum and CSF levels of IL-6, ET-1, and S100B. TCD was conducted daily from days 3 to 10, and suspected vasospasm was confirmed using CT angiography. The demographic, clinical, and radiological data were recorded. SIBM SPSS Statistics for Windows, Version 24 (Released 2016; IBM Corp., Armonk, New York) was used to conduct the statistical tests, and the t-test or Mann-Whitney U test was used to compare the continuous variables. The biomarker thresholds were determined through receiver operating characteristic (ROC) curve analysis, and the independent predictors of vasospasm were identified using logistic regression models. Statistical significance was set at p < 0.05.

The Institutional Review Board of Bacha Khan Medical College, Mardan, Pakistan, approved the study protocol (Ref no. 2188/BKMC). All participants or their legal representatives signed informed consent forms to participate in the study, in accordance with the Helsinki Declaration. The inclusion criteria for the study were patients 18 years of age or older with aSAH confirmed by CT angiography that presented within 48 hours after ictus. The exclusion criteria excluded patients with traumatic subarachnoid hemorrhage, chronic inflammatory or autoimmune diseases, or severe liver or kidney dysfunction, and those who refused to participate.

The demographic and clinical data were obtained using electronic medical records. The levels of the biomarkers were recorded on specific sampling days. Vasospasm was diagnosed based on TCD velocities and the CT angiography findings. The results were vasospasm, the Glasgow Outcome Scale (GOS) at discharge, the data on ICU stays, and mortality.

The statistical analysis was performed using SPSS 24.0 software. The descriptive statistics (mean, standard deviation (SD), or median (interquartile range, IQR)). T-tests were used to compare the groups. Logistic regression was used to identify independent predictors of vasospasm. The biomarker cutoffs were determined using ROC curves. The level of significance was p < 0.05.

## Results

**Figure 2:**
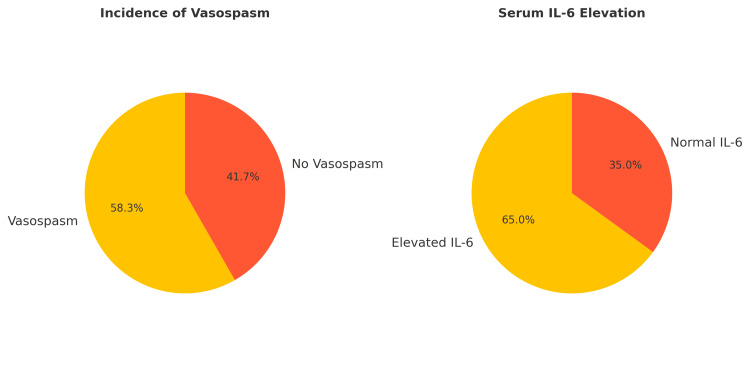
Vasospasm incidence (58.3%) and of IL-6 elevation in non-vasospasm patients (65.0%). IL: interleukin

This study analyzed 60 patients with a mean age of 55.4 ± 12.1 years, of whom 38 (63%) were female. Vasospasm was radiologically confirmed in 35 (58%) of the patients, most frequently occurring on day 5 (IQR 4-7). The demographic and clinical characteristics of the vasospasm and non-vasospasm groups were similar, as shown in Figure 2. The numerical values are presented in Tables 2, 3. Key differences were observed in the biomarkers, with significant elevations in the serum and CSF levels of IL-6 and ET-1 in the vasospasm group, suggesting the potential role of these biomarkers in predicting vasospasm and clinical outcomes.

**Table 2:** Analyses of the patient demographic and clinical characteristics and continuous variables (age) using the independent samples t-test and of the categorical variables using Pearson's chi-square test. *Independent t-test. ^†^Chi-square test. SD: standard deviation.

Patient Demographics	Variable Vasospasm Group (n = 35)	No Vasospasm Group (n = 25)	Test Statistic	p-value
Age (years), mean ± SD	55.8 ± 11.2	54.3 ± 12.8	t = 0.36*	0.72
Gender (female), n (%)	22 (62.9%)	16 (64.0%)	χ² = 0.01†	0.91
Hunt & Hess Grade ≥ 3, n (%)	18 (51.4%)	10 (40.0%)	χ² = 0.75†	0.38
Hypertension, n (%)	20 (57.1%)	13 (52.0%)	χ² = 0.16†	0.68
Diabetes mellitus, n (%)	6 (17.1%)	4 (16.0%)	χ² = 0.02†	0.88

**Table 3:** All of the biomarkers analyzed with the independent samples t-test. *p<0.05, **p<0.01, **p<0.001. IL: interleukin; CSF: cerebrospinal fluid; ET: endothelin; S100B: S100 calcium-binding protein.

Biomarker	Vasospasm Group (n = 35)	No Vasospasm Group (n = 25)	Test Statistic	p-value
Serum IL-6 (pg/mL)	124.8 ± 45.7	72.3 ± 30.4	t = 4.83*	0.002
CSF IL-6 (pg/mL)	456.2 ± 120.5	290.1 ± 98.4	t = 5.62*	0.001
Serum ET-1 (pg/mL)	3.8 ± 1.2	2.1 ± 0.9	t = 5.02*	0.01
CSF ET-1 (pg/mL)	12.6 ± 4.3	7.4 ± 3.1	t = 4.67*	0.008
Serum S100B (pg/mL)	0.42 ± 0.15	0.34 ± 0.13	t = 1.79*	0.08

Key findings

The study revealed significant differences in biomarker levels between the patients who developed vasospasm and those who did not. On day 3, the mean serum IL-6 levels were markedly higher in the vasospasm group (124.8 ± 45.7 pg/mL) than in the non-vasospasm group (72.3 ± 30.4 pg/mL; p = 0.002). A similar trend was observed in the CSF, where IL-6 levels were significantly elevated in the vasospasm patients (456.2 ± 120.5 pg/mL compared with 290.1 ± 98.4 pg/mL; p = 0.001). The ET-1 levels also increased markedly in the vasospasm group in both the serum (3.8 ± 1.2 pg/mL compared with 2.1 ± 0.9 pg/mL; p = 0.01) and the CSF (12.6 ± 4.3 pg/mL compared with 7.4 ± 3.1 pg/mL; p = 0.008). While S100B levels showed no significant upward trend, they remained elevated in the vasospasm patients on day 7, though at levels lower than earlier peaks.

Further analysis identified serum IL-6 levels greater than 100 pg/mL on day 3 (95% CI 1.40-7.40; p = 0.005) and CSF ET-1 levels greater than 10 pg/mL (OR 2.8; 95% CI 1.21-6.67; p = 0.015) as independent predictors of vasospasm. The ROC analysis demonstrated strong predictive value for serum IL-6, with an area under the curve of 0.81 (95% CI 0.70-0.91) and an optimal cutoff of 98 pg/mL (79% sensitivity, 75% specificity), as shown in Table 4.

**Table 4:** Logistic regression and ROC analysis. IL: interleukin; CSF: cerebrospinal fluid; ET: endothelin; CI: confidence interval; ROC: receiver operating characteristic.

Biomarker (Day 3)	95% CI	p-value
Serum IL-6 > 100 pg/mL	1.4–7.4	0.005
CSF ET-1 > 10 pg/mL	1.2–6.6	0.015

Clinically, elevated serum IL-6 (>98 pg/mL) on day 3 was associated with poorer outcomes, as reflected in lower GOS scores (mean 2.8 ± 0.9 compared with 4.1 ± 0.7; p = 0.013). However, no significant differences were observed in the length of stay in the intensive care unit (ICU) or in survival, although biomarker-positive patients exhibited a trend toward earlier intervention. These findings suggest that IL-6 and ET-1 may serve as valuable early indicators of vasospasm risk and poor neurological outcomes.

## Discussion

Diagnosing CV following aSAH is an important step toward improving patient outcomes because CV causes delayed cerebral ischemia and subsequent lifelong neurological deficits [15]. We have shown that increased concentrations of IL-6 and ET-1 in the serum and CSF on day 3 after ictus were associated with the subsequent occurrence of vasospasm [16]. These results are consistent with and add to the existing research on inflammatory and endothelial markers in neurovascular pathology. One pro-inflammatory cytokine, IL-6, has long been studied in terms of its role in the post-hemorrhagic inflammatory process. The first evidence that levels of IL-6 in the CSF rise significantly in patients who develop vasospasm following aSAH was reported in earlier investigations [17]. This evidence was also reinforced by findings that an increase in IL-6 was associated with the severity of vasospasm and adverse clinical outcome [18]. Our results support the use of IL-6 as an early biomarker, for both serum and CSF IL-6 levels increased significantly in the vasospasm group as early as day 3, indicating a potential diagnostic and treatment window before the onset of clinical or radiographic changes. Likewise, ET-1, another potent vasoconstrictor peptide produced in endothelial cells, has been reported to aggravate the pathophysiology of cerebral vasospasm. McGuire et al. found that increased concentrations of ET-1 in CSF and plasma preceded angiographic vasospasm and concluded that ET-1 may act not only as a marker but also as a therapeutic target [19]. We likewise found that patients with greater than 10 pg/mL of ET-1 in their CSF were much more likely than patients with lower levels to suffer vasospasm, and this finding suggests that elevated levels of ET-1 are predictive. Surprisingly, the vasospasm group did not show a significant difference from the control in terms of S100B levels, which increase in cases of glial activation and injury. This ambiguity was observed by Herrmann et al., who reported that S100B levels increased after aSAH and were not consistently associated with the development of vasospasm [20]. The ambiguity could indicate the non-specificity of S100B or the fact that it correlates with brain injury at a global level rather than with local vasospastic change. Our results are also consistent with those of a meta-analysis that emphasized the greater diagnostic value of aggregated sets of biomarkers compared with single-biomarker analyses, especially in cases involving multifactorial pathogenesis such as vasospasm [21]. The multimarker combination of IL-6 and ET-1 enhanced the predictive performance of our regression model, and this result similarly suggests that multimarker strategies may be superior.

Furthermore, we found that high levels of IL-6 tended to correlate with low GOS scores at discharge, with earlier concentrations of IL-6. This finding substantiates an earlier report that high IL-6 levels predicted not only vasospasm but also long-term cognitive impairment and dependence [22]. It is possible that IL-6 plays a role in vasospasm, leading to further neuroinflammatory harm. Although angiographic and TCD techniques remain the gold standard for diagnosing vasospasm, they are frequently reactive. Conversely, biomarkers may predict pathophysiological changes days before they occur. One previous study suggested that preemptive therapeutic measures, such as up-titration of nimodipine or endovascular treatment, may be possible with serial biomarker monitoring of high-risk patients [23]. We found validation for this model in that our work defined biomarker thresholds on day 3 that can be used to predict vasospasm.

The small sample size and single-center design of this study are limitations that may reduce its generalizability. Furthermore, we did not investigate genetic polymorphisms that could affect individual expression of the biomarkers, although increased susceptibility to vasospasm may be modified by genetic variants in cytokine or endothelial pathways [24]. Additional study is needed to take into consideration genomic and proteomic assessments for better biomarker-based prediction models.

Moreover, cost and time, as well as inconsistency in the assay method, complicate the incorporation of biomarker data into a clinical protocol. Before clinical application, standardization of thresholds and sampling time is necessary [25]. Nevertheless, our study offers strong evidence that serum and CSF biomarkers, especially IL-6 and ET-1, can be used for the early detection and risk stratification of cerebral vasospasm after aSAH.

Several other limitations should be considered when interpreting these findings. Again, the moderate sample size may have affected the statistical power to detect small but clinically relevant effects. Second, while we accounted for major demographic and clinical variables, residual confounding remains a potential limitation because of unmeasured factors such as medication adherence and genetic predispositions. Lastly, as mentioned, the single-center design, although ensuring standardized protocols, introduces a limitation regarding generalizability to broader populations. These limitations highlight the need for validation of the findings presented here in larger multicenter studies.

## Conclusions

This study shows significant associations of elevated IL-6 and ET-1 levels in both serum and CSF with the occurrence of vasospasm, suggesting the potential role of IL-6 and ET-1 as biomarkers for risk stratification. While demographic factors did not differ between the groups, the biochemical findings demonstrate the importance of inflammatory and endothelial pathways in vasospasm pathogenesis. The clinical implications of this study include the potential use of these biomarkers for the early identification of patients at high risk for cerebral vasospasm and for revealing targets for future therapeutic interventions. Although further validation is needed, these results contribute evidence useful for the development of personalized monitoring strategies in neurovascular care, thereby helping to bridge a critical gap between laboratory findings and bedside application.
